# Treatment of Calcinosis in Dermatomyositis—Case Report and Review

**DOI:** 10.3390/jcm13206234

**Published:** 2024-10-18

**Authors:** Alicja Frączek, Jakub Kuna, Joanna Rybak d’Obyrn, Magdalena Krajewska-Włodarczyk, Agnieszka Owczarczyk-Saczonek

**Affiliations:** 1 Department of Dermatology, Sexually Transmitted Diseases and Clinical Immunology, Collegium Medicum, University of Warmia and Mazury, Al. Wojska Polskiego 30, 10-229 Olsztyn, Poland; 2 Department of Rheumatology, Collegium Medicum, University of Warmia and Mazury, Al. Wojska Polskiego 30, 10-229 Olsztyn, Poland

**Keywords:** autoimmune diseases, calcinosis cutis, dermatomyositis, intralesional sodium thiosulfate, intravenous immunoglobulins

## Abstract

**Background/Objectives:** Calcinosis cutis (CC) is a condition that may develop in the course of several autoimmune connective tissue diseases (ACTDs). Among these, the conditions most frequently associated with CC are systemic sclerosis (SSc) and dermatomyositis (DM). Despite both the prevalence and diversity of available treatment options, therapeutic recommendations remain not fully established due to a limited number of studies and lack of unambiguous evidence regarding their effectiveness. **Case Presentation:** We report two cases of patients with DM and concomitant massive cutaneous calcifications who were treated: in the case of a 71-year-old man with DM and past medical history of primary cutaneous T-cell lymphoma (CTCL) who received intralesional (IL) 25% sodium thiosulfate (STS) with platelet-rich plasma (PRP) injections, and, in the case of a second patient, 24-year-old woman with nephrolithiasis, who received intravenous immunoglobulin (IVIG) infusions at a dose of 2 g/kg in combination with prednisone at a dose of 5 mg/day. **Conclusions:** The applied treatment led to reduction in pain, size, and number of calcified lesions. Additionally, healing of fingertip ulcers after PRP injections was observed. While this report highlights only two isolated cases, the use of IVIG and STS with PRP injections appears to be an effective treatment method. Nevertheless, both standardization and additional research are required.

## 1. Introduction

Calcinosis is a common complication of systemic autoimmune diseases, the pathogenesis of which remains not fully understood [1,2]. Typically, insoluble agglomerates of calcium phosphate salts may occur in the form of nodules or plaques that tend to accumulate in the skin, subcutaneous tissue, fascia, muscles or blood vessels [3]. Thus far, few theories have been proposed to account for the development of calcinosis. One of these suggests microvascular changes as a potential explanation of calcinosis cutis (CC) formation, which is supported by the observed associations between calcinosis and digital ischemia among patients with systemic sclerosis (SSc) [4]. Another theory is associated with dysregulation of the innate immune system. A proposed mechanism is based on the potential similarity between CC and vascular calcinosis formation provoked i.a. by proinflammatory cytokines, such as IL-1, IL-6 and TNF-α, as well as reactive oxygen species (ROS) production among patients with juvenile DM [1]. Additionally, a correlation with specific autoantibodies, including melanoma differentiation-associated gene 5 antibody (MDA5), autoantibody to nuclear protein matrix 2 (anti-NXP2 antibody) anti-mitochondrial antibodies (AMA), anti-centromere antibody (ACA) or even antinuclear antibodies (ANA), has also been described in some studies analyzing the pathogenesis of CC in autoimmune diseases [5,6,7,8].

Soft tissue calcifications can be divided into four main groups [9], with dystrophic calcinosis being the most frequent [10]. This classification further differentiates idiopathic, metastatic and iatrogenic forms [11]. Additionally, some authors distinguish a fifth category, calciphylaxis, also known as calcium-uremic arteriolopathy, which is a potentially life-threatening condition [12]. Thus far, calcinosis has been documented among patients with dermatomyositis (DM) [13], juvenile dermatomyositis (JDM) [14], polymyositis (PM) [15], systemic sclerosis (SSc) [16], systemic lupus erythematosus (SLE) [17], mixed connective tissue disease (MCTD) [18], Sjögren’s syndrome [19] and rheumatoid arthritis [20]. However, SSc and DM are considered the most frequently associated with CC [21]. Publications indicate that calcinosis can affect approximately 30% of patients with DM, particularly the juvenile form [22], and even in 18–49% of patients with SSc [23,24]. Nevertheless, according to some reports, up to 50% of CC may not be detected during a standard physical examination at an early stage of the disease [25]. The most commonly observed clinical effects include contractures, ischemia, muscle atrophy, itching and ulcers [26], which significantly impact patients’ quality of life and may require a multidisciplinary approach.

Treatment options encompass both pharmacological and non-pharmacological strategies, with the specific choice tailored to factors such as size, number and location of lesions, as well as presence of concurrent inflammation [27]. Currently, calcium channel blockers, including diltiazem or amlodipine, represent the most commonly employed pharmacological interventions [12,28,29]. Bisphosphonates [30] or colchicine [31,32], due to their anti-inflammatory properties, are further alternatives, although evidence supporting their efficacy remains limited [9]. In 2004, sodium thiosulfate (STS) was first reported as a potential treatment for CC [33]. Its ability to increase calcium’s solubility and its relatively small number of potential side effects, including mainly skin irritation and pain during the injection procedure, led to its application in the management of calcinosis [34]. The currently available publications highlight beneficial effects of applied STS in topical and intralesional forms. Topical therapy is the most successful for lesions up to 0.2 cm in size, while intradermal therapy is more effective for lesions up to 2.0 cm [35]. Intravenous administration seems to be a less beneficial approach, with a greater risk of adverse effects [36]. Another therapeutic option, minocycline, has been shown to be effective in reducing calcium deposits and promoting ulcer healing [37,38,39]. While a single case report suggests the potential benefits of ceftriaxone for calcinosis in morphea, further research is needed [40]. Additionally, cutaneous ulcerations, affecting 3–19% of patients with DM [41] may be successfully treated with the use of PRP. In a pilot study published in 2016 that included 20 patients with ulcer vasculitis, two of whom with DM, the use of PRP in conjunction with an occlusive dressing containing 10% calcium chloride resulted in the full healing of the ulcers within six weeks [42]. Further treatment possibility includes intravenous immunoglobulins (IVIG), which, probably due to their potential to reduce macrophages and cytokines, may also prove beneficial effects among patients with DM. Nevertheless, thus far published research results are contradictory [43,44,45,46]. Among other therapeutic options it can be distinguished treatment with tumor necrosis factor-α inhibitors (TNFi) such as infliximab [47,48], Janus kinase inhibitors (JAKi) such as tofacitinib [49] or B cell-depleting monoclonal antibodies (e.g., rituximab) [50]. If the effects of pharmacological treatment are not satisfactory, surgical methods can be considered in some cases [51,52,53], nevertheless, their clinical relevance has been declining in recent years. As alternatives, extracorporeal shock wave lithotripsy (ESWL) [54,55] or laser therapy [56] can be performed. Despite the universality of calcinosis and the availability of treatment methods, it remains a therapeutic challenge. To expand on potential treatment strategies, here, we present two cases of patients with DM and CC who were successfully treated with IL 25% STS with PRP injections and IVIG.

## 2. Case Report

### 2.1. Case One

A 71-year-old man with DM and past medical history of primary cutaneous T-cell lymphoma (CTCL, Mycosis Fungoides form) was admitted in March 2023 to the Clinic and Department of Dermatology, Sexually Transmitted Diseases and Clinical Immunology of the Municipal Hospital in Olsztyn, Poland, to undergo treatment with IL STS and PRP injections due to the presence of fingertip ulcerations and calcinosis. The first non-specific skin lesions appeared in 2000. In 2013, after histopathological examination, immunophenotyping of peripheral blood and hematology consultation, CTCL was diagnosed. At that time, PUVA and, later, due to psoralen intolerance, UVB phototherapy from March 2013 to January 2014; interferon α (INF-α) from March 2014 until the turn of 2014/2015; and bexarotene treatment from July 2014 to July 2015 were applied. One year later, after the CTCL diagnosis, the patient started to report periodic muscle weakness, swelling of the face and numbness with tingling in the fingers. Subsequently, papules above the metacarpophalangeal and interphalangeal joints on hands (called Gottron papules) and calcinosis of the armpits, thighs and buttocks in the projection of the ischial tuberosities and abdomen appeared. After correlating the laboratory and histopathological results with the patient’s clinical features and meeting the Bohdan and Peter classification criteria [57], in 2014, a diagnosis of Wong-type DM was made. Treatment for DM included IVIG from August to November 2019, which, due to the significant severity of muscle and skin symptoms, was reintroduced again in 2021 from October to December, with once per month infusions at a dose of 2 mg/kg. Methotrexate (MTX), which was administered at a dose of 12.5 mg/week from 2019, with later reduction of a dose to 7.5 mg/week in conjunction with prednisone at a dosage of 10–15 mg/day. In 2022, a deterioration of the clinical condition was observed. Finally, treatment with the use of MTX was completed in 2023. In addition, mycophenolate mofetil (MMF) in a dose of 1000 mg/day was initiated from May and was terminated in August 2022 due to adverse reactions. Later, due to mediocre clinical improvement, from September 2022 to April 2023, therapy with rituximab (RTX) at a dose of 1 g i.v. was applied (in total 4 doses, 6 months after the initial treatment). Additionally, hydroxychloroquine at a dose of 200 mg/day was implemented from the moment of initiation of rituximab. Next, a series of STS and PRP injections were introduced (Table 1).

**Scheme 1:**
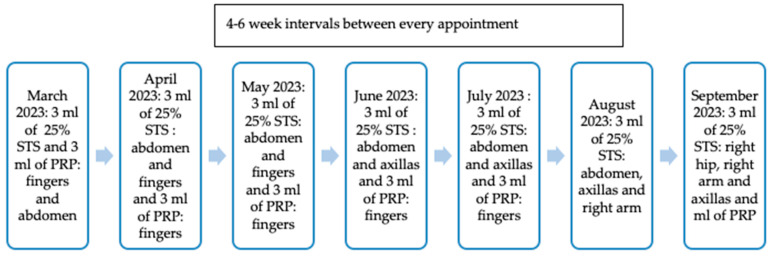
Strategy diagram, including the schedule of appointments with the treatment areas and applied methods.

**Figure 1:**
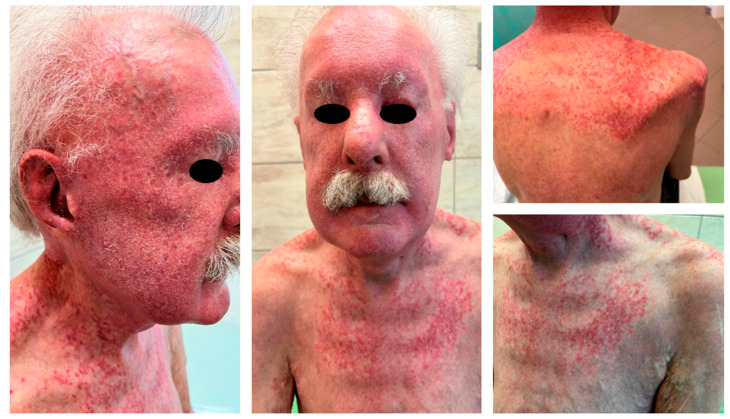
Clinical presentation of the patient at first visit in March 2023.

**Figure 2:**
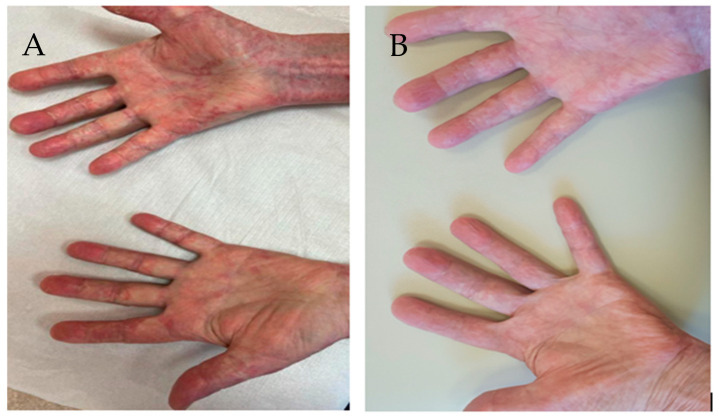
Patient’s hands (palmar side) before (**A**) and after 7 months of treatment (**B**) with intralesional 25% sodium thiosulfate and platelet-rich plasma injections.

**Figure 3:**
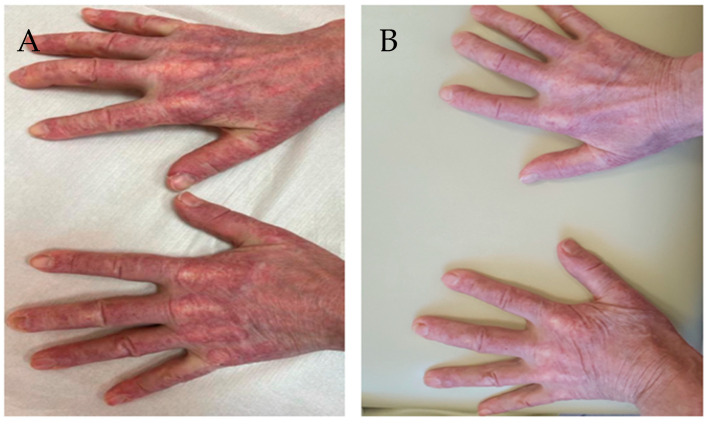
Patient’s hands (dorsal side) before (**A**) and after 7 months of treatment (**B**) with intralesional 25% sodium thiosulfate and platelet-rich plasma injections.

**Figure 4:**
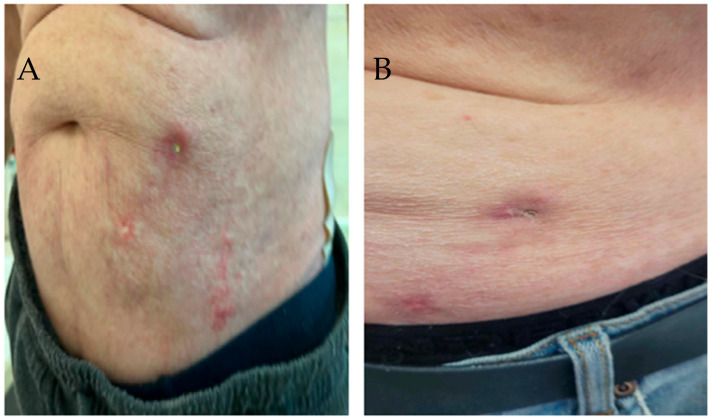
Patient’s abdomen before (**A**) and after 7 months of treatment (**B**) with intralesional 25% sodium thiosulfate injections.

**Figure 5:**
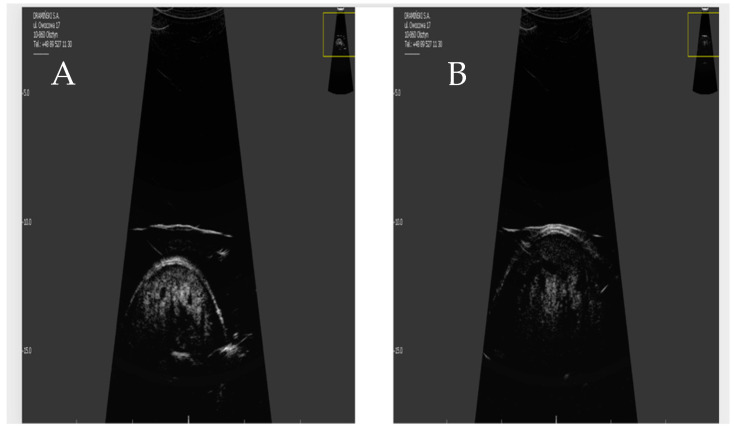
Ultrasonography image of the calcium deposit of the patient’s fingertip before (**A**) and after 7 months of treatment (**B**) with intralesional sodium thiosulfate and platelet-rich plasma injections.

On the day of admission to the clinic, confluent erythematous-papular lesions with a vascular component were present on the patient’s scalp, face, neck, upper limbs, upper back and lumbar area (Figure 1). Additionally, inflammatory changes at the edges of the eyelids, with ectropion and thinned skin on the fingertips, along with small scars and vascular lesions, were visible (Figure 2). In the subcutaneous tissue around the armpits and the lateral surface of the thighs, the buttocks in the projection of the ischial tuberosities and on the lower abdomen, nodules and flat tumors were palpable. The laboratory results showed a presence of ANA Hep-2: 1:320, immunoblot (IB) ANA profile: Ro25^+++^, IB myositis-Ro52^+++^. The treatment included IL 25% STS and PRP injections of the fingertips and lesions on the abdomen and armpits. Seven appointments were scheduled from March until the end of September 2023, with approximately once-a-month frequency (Scheme 1). Apart from pain during the procedure resulting from the injections, no other side effects were noted. Due to surgical treatment for ectropion, which occurred to be caused by 1.5 cm-long calcium deposits, further injecting procedures were temporarily halted. Nevertheless, the implemented therapy led to a reduction in the number of calcified lesions located on the fingers, armpits and abdomen, with decrease in pain and healing of the fingertip ulcers (Figure 2, Figure 3, Figure 4 and Figure 5). Therefore, it seems to be effective, easy and relatively cheap method. 

### 2.2. Case Two

A 24-year-old woman with long-term history of DM and kidney stone disease was admitted to the Rheumatology Clinic of the Municipal Hospital in Olsztyn, Poland, in January 2022 due to progressive weakness and numerous oozing calcified skin lesions of the upper and lower limbs. The first symptoms of the disease, in the form of enlargement of the circumference of the right thigh with coexisting calcifications, were reported in 2001 when the patient was three years old. Due to the increasing size and limited mobility of the right leg, a surgical procedure was performed in 2005. Histological analysis of the removed tissue showed caseous masses, prompting anti-tuberculosis therapy. In 2009, a diagnosis of JDM was established, and then treatment with prednisone was applied. Three years later, the patient experienced further symptoms, including muscle weakness and purulent inflammation of the right elbow joint. The electromyographic examination (EMG) revealed features of primary muscle damage, suggesting a diagnosis of post-steroid myopathy. Consequently, from November 2012 to January 2013, IVIG infusions were administered, with later attempts to implement therapies with MTX at a dosage of 25 mg/week, cyclosporine (CsA) and pamidronate (PMD) over the course of 4 years (Table 1). Additionally, numerous surgical procedures involving the removal of calcinosis deposits and the enhancement joint mobility were performed. From 2016, after turning 18 years old, until 2022, the patient remained under general practitioner care and did not attend follow-up visits. Throughout that time, only prednisone therapy was administered.

**Scheme 2:**
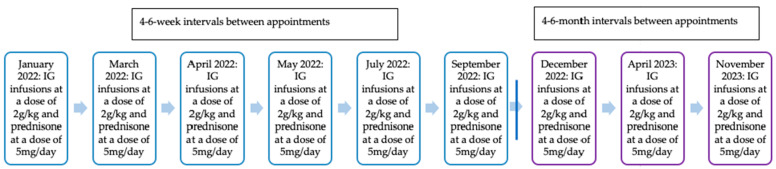
Strategy diagram including the schedule of appointments with the doses of applied treatment.

**Figure 6:**
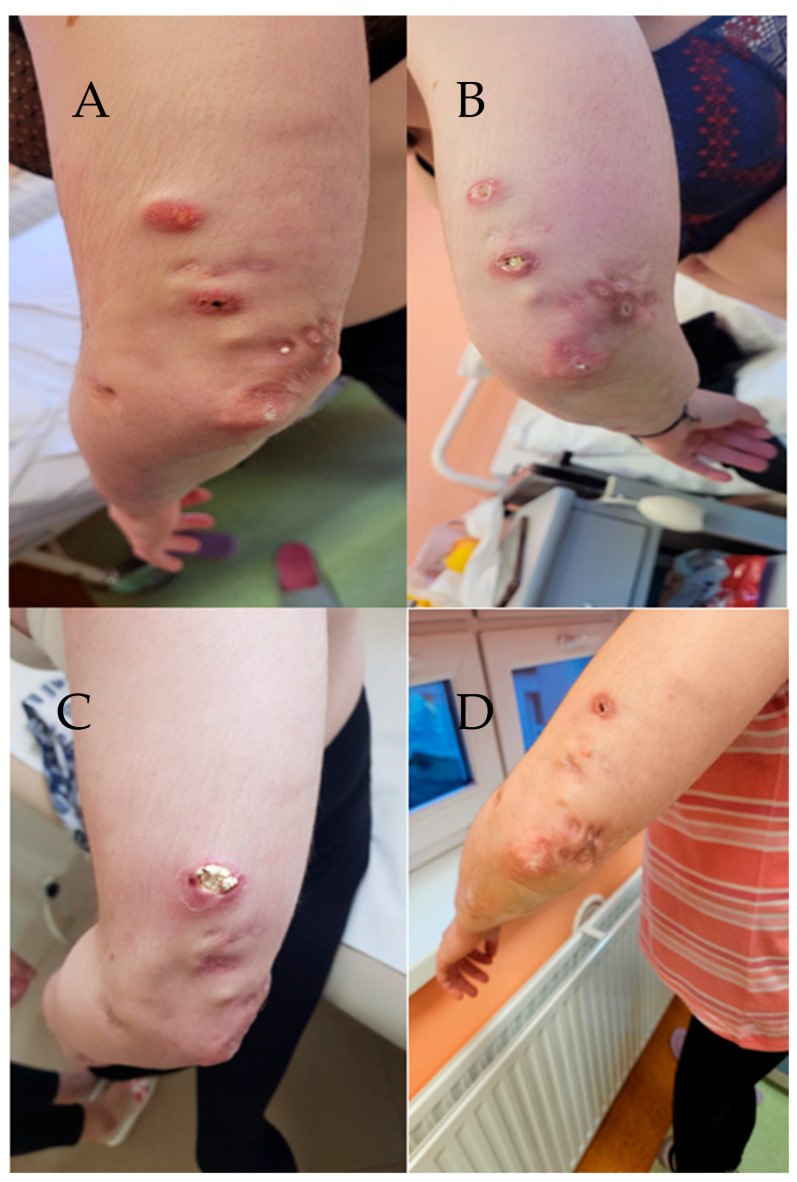
Calcium lesions in the left upper limb during the patient’s hospitalization on the day of admission in January 2022 (**A**), 3 months (**B**), 6 months (**C**) and 1 year and 4 months (**D**) after implementation of intravenous immunoglobulin treatment.

**Figure 7:**
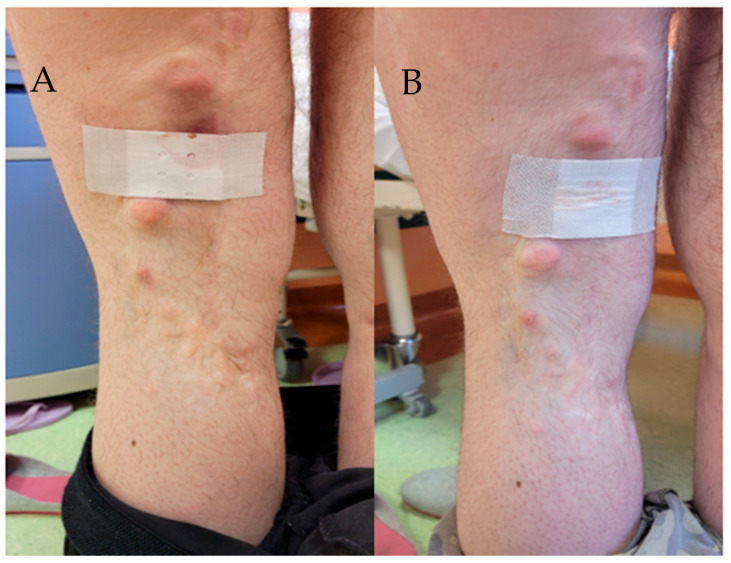
Calcium lesions in the patient’s lower limbs on the day of admission in January 2022 (**A**) and 3 months (**B**) after implementation of intravenous immunoglobulins treatment.

**Figure 8:**
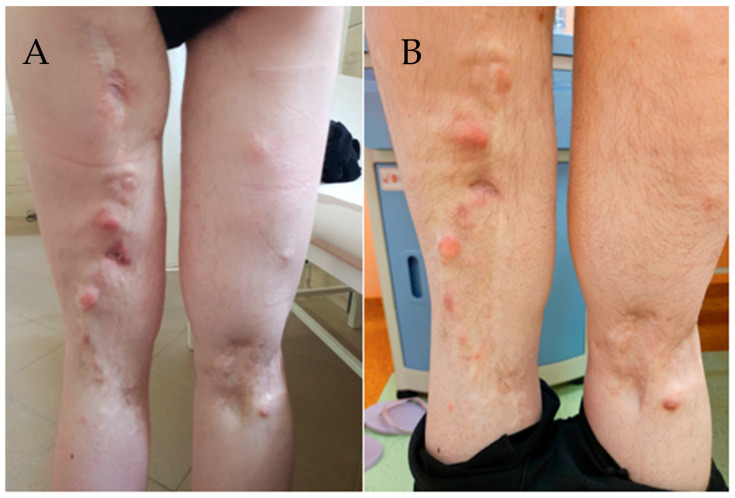
Calcium lesions in the patient’s lower limbs 9 months (**A**) and 1 year and 4 months (**B**) after implementation of intravenous immunoglobulins treatment.

**Figure 9:**
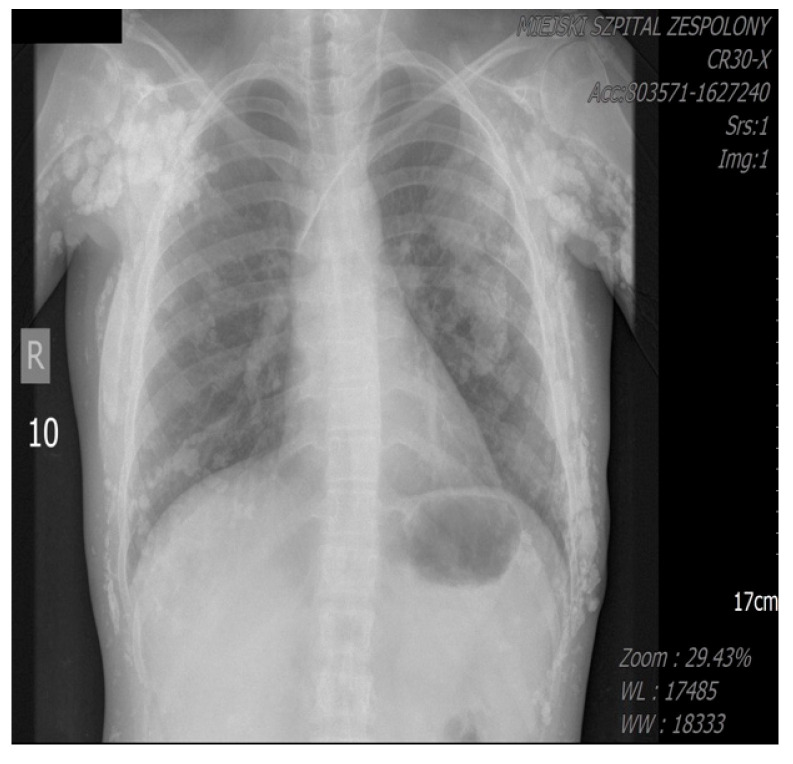
Chest X-ray during the patient’s hospitalization in January 2022, before the implementation of immunoglobulin treatment. Numerous calcifications present in the subcutaneous tissue of the chest.

**Figure 10:**
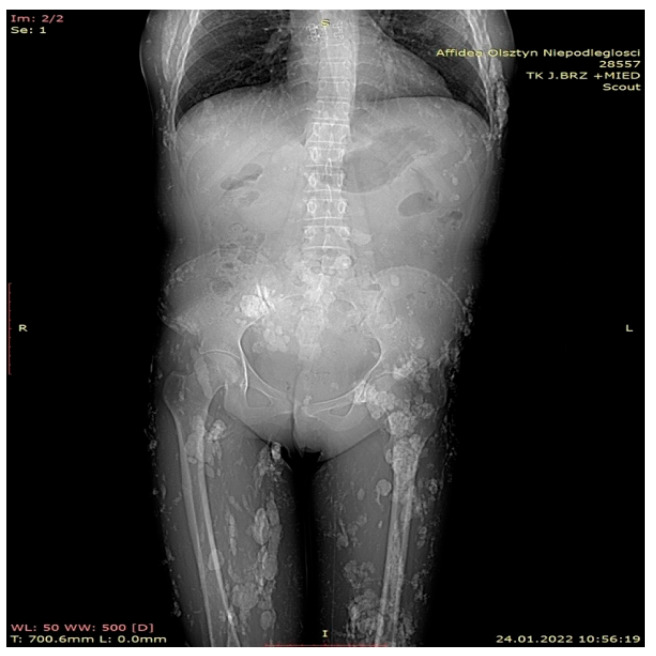
Coronal computed tomography scan of the abdomen and pelvis during the patient’s hospitalization in January 2022, before the implementation of immunoglobulin treatment. Visible calcifications in the abdominal cavity and pelvis, most frequently in the lower limbs.

**Figure 11:**
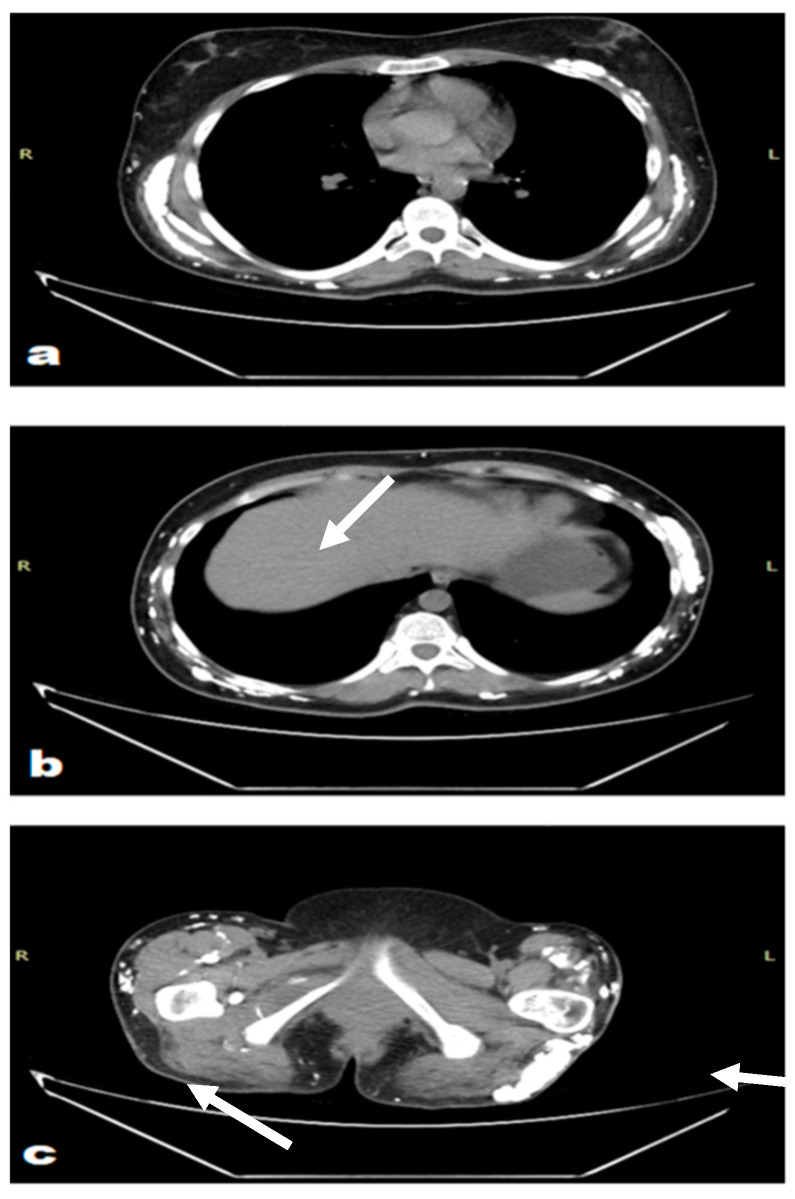
Scans before the implementation of immunoglobulin treatment in the transverse plane of the computed tomography examination: (**a**,**b**) at the level of the chest (**c**) at the level of the buttocks. Arrows mark larger areas of subcutaneous calcifications.

On the day of admission to the clinic, physical examination revealed hirsutism, limited mobility of the elbow, shoulder and hip joints, muscle atrophy and numerous calcium nodules in the skin of the abdomen, chest, upper and lower limbs, some with inflammation (Figure 6, Figure 7 and Figure 8). In laboratory results, microcytic anemia was observed, with reduced levels of iron, ferritin and UIBC. Chest X-ray and computed tomography (CT) scan of the abdomen and pelvis revealed extensive areas of calcification (Figure 9, Figure 10 and Figure 11). During hospitalization, treatment with IG infusions at a dose of 2 g/kg with prednisone at a dose of 5 mg/day on a 2-day protocol was administered. The first six doses of IVIG were administered at 1–1.5-month intervals, with later extension between doses to 4–6 months. In total, nine courses of IVIG infusions were performed (Scheme 2). Apart from two reported migraine-type headaches during all procedures, the patient did not experience any other adverse events. Due to the implementation of therapy, a reduction in the number of calcified lesions and inhibition of the formation of new ones were observed (Figure 6, Figure 7 and Figure 8).

## 3. Discussion

Currently, there are no established guidelines regarding the treatment of patients with calcinosis in autoimmune connective tissue diseases. Despite numerous treatment strategies, including pharmacological and nonpharmacological methods, calcinosis remains a challenge. A limited number of reported cases have investigated STS injections as a treatment method for CC [58,59,60,61]. Several case studies have described topical [62] and intravenous [63] use of STS as a therapeutic option for calcinosis; however, both methods have limitations, including the extent of skin lesions and potential adverse effects such as nausea, vomiting or headache, mainly associated with STS infusion [64]. In presented clinical case, IL injections of STS and PRP reduced the number of calcification lesions on the fingers and armpits. Moreover, pain relief and healing of fingertip ulcers were achieved. Therefore, it seems that IL STS and PRP injections can be an effective, easy and comparably cheap treatment option. Similar observations were reported in a pilot study describing PRP combined with a calcium chloride dressing as an effective method for healing cutaneous ulcers, including those presented among patients with DM [42]. Moreover, according to the available publications, therapeutic methods based on topical and intradermal STS have been successfully used for the treatment of CC with very few or no adverse effects [34]. The positive results of STS may be explained by its anti-inflammatory and antioxidant properties as well as its calcium chelation capacity [35]; however, the effectiveness of the applied form of STS depends, among other factors, on the size of the lesion being treated. Lesions up to 0.2 cm in size respond the best to topical therapy, while lesions up to 2.0 cm can be treated more successfully with intradermal injections [35]. The observations reported in that case are consistent with the results published by Goossens et al. [65], where the positive effects of IL STS injections were noticed after 12 and 21 months of weekly repeated procedures in a patient with DM and a patient with familial tumoral calcinosis. Further, a case series of five patients with CC demonstrated successful treatment with IL STS, confirmed by follow-up in ultrasound imaging [66]. In contrast, in a double-blind, placebo-controlled study utilizing IL STS with 1- and 2-month follow-ups of four patients, there was observed improvement in the size and Physician Global Assessment score of the lesion in only one patient. While no statistically significant difference in mean lesion size between treatment and control groups was observed at the 3-month follow-up, the results are limited by the small group size, single-center design, and relatively short study duration [67].

The effectiveness of intravenous immunoglobulin in the treatment of calcinosis has been documented in a few studies. It is hypothesized that intravenous immunoglobulin (IVIG) therapy may influence the progression of calcinosis cutis (CC) by exerting anti-inflammatory effects, possibly through the inhibition of activated macrophages [27,68]. Despite reports of the beneficial effects of IVIG therapy in reducing calcium deposits [44,69,70,71], the available data are contradictory [45]. In a study with eight patients who received IVIG infusions to treat refractory cutaneous disease with calcinosis and both refractory cutaneous and muscle disease with calcinosis, improvement was observed in five of eight patients treated with IVIG, whereas the rest demonstrated stable or worsened disease [72]. In turn, in a case published by Schanz et al., administration of IVIG treatment for a 53-year-old woman with severe calcium deposits led to the full disappearance of calcinosis within 14 cycles of the treatment [69]. In another case, a 55-year-old female patient who had not responded to previous therapies was given IVIG at a dose of 0.4 g/day, together with a decreased dose of prednisone. After five cycles of treatment, healing of ulcers and a reduction of calcification lesions were observed. The patient has continued to receive maintenance therapy with a yearly course of IVIG without prednisone for five years of follow-up. During that time, skin condition remained well-controlled [46]. Presented in this publication case indicates successful use of IVIG in a patient with DM and coexisting extensive areas of calcinosis. The applied therapy led to a decrease in skin lesions and inhibition of the formation of new ones. The observed clinical response suggests a beneficial effect of IVIG treatment on DM symptoms and a reduction in calcium agglomerates observed in the course of calcinosis cutis. Nevertheless, only one case with the successful use of the above-mentioned method is presented in this study.

## 4. Conclusions

While this report highlights two isolated cases, it seems that both therapies, including intravenously applied IVIG and IL STS with PRP injections, might yield satisfactory clinical results. Even though treatment based on the above-described methods among patients with calcinosis in the course of autoimmune connective tissue diseases is still not well established, it can be considered in the therapeutic plan. Due to that appropriate evaluation of all the advantages and potential side effects, as well as, the features of treated lesions in order to achieve the best possible clinical results is required. Therefore, further research and standardization of both procedures are needed.

## Figures and Tables

**Table 1:** Demographic data and applied treatment in the course of dermatomyositis in patients.

Patient	Sex	Age (Years)	Comorbidities	Clinical Features of DM	Applied Treatment (with Doses) in the course of DM	Dates (Years)
Patient 1	M	71	Hepatosteatosis, vitamin B12 deficiency, gastropathy, hiatal hernia, sigmoid diverticula, hypertension, hyperlipidemia, CTCL in remission, right-sided inguinal hernia operation (2020), prostatectomy due to prostate cancer (2014), cataract surgery and posterior vitrectomy (2014)	Periodic muscle weakness, swelling of the face and numbness with tingling in the fingers. Gottron’s papules, calcinosis, ectropion, thinned skin on fingertips with ulcers	Intravenous immunoglobulin 2 mg/kg	2019–2021 and 2022–2022
					Methotrexate 12.5 mg/week with prednisone 10–15 mg/day	2019–2022
					Rituximab 1 g with hydroxychloroquine 200 mg	2022–2022
					Mycophenolate mofetil 1 g/day	2022–2022
					Intralesional 25% sodium thiosulfate with platelet-rich plasma 3 mL	2023–2023
					Surgical operation of ectropion	2023–2024
Patient 2	F	24	Nephrolithiasis, hepatosteatosis, steroid-induced glaucoma	Progressive weakness, calcinosis	Methotrexate 25 mg/week	2013–2013 and 2014–2016
					Cyclosporine (unknown dose)	2013–2014
					Pamidronate (unknown dose)	2013–2016
					Intravenous immunoglobulin 2 mg/kg	2013 and 2022–2023
					Prednisone 5 mg/day	2016–2024

## Data Availability

The original contributions presented in the study are included in the article, further inquiries can be directed to the corresponding author.
